# The strength of a remorseful heart: psychological and neural basis of how apology emolliates reactive aggression and promotes forgiveness

**DOI:** 10.3389/fpsyg.2015.01611

**Published:** 2015-10-27

**Authors:** Urielle Beyens, Hongbo Yu, Ting Han, Li Zhang, Xiaolin Zhou

**Affiliations:** ^1^Center for Brain and Cognitive Sciences, Peking University, Beijing, China; ^2^Department of Psychology, Peking University, Beijing, China; ^3^Key Laboratory of Machine Perception (Ministry of Education), Peking University, Beijing, China; ^4^Beijing Key Laboratory of Behavior and Mental Health, Peking University, Beijing, China; ^5^PKU-IDG/McGovern Institute for Brain Research, Peking University, Beijing, China

**Keywords:** apology, forgiveness, reactive aggression, Implicit Association Test, ERP, LPP

## Abstract

Apology from the offender facilitates forgiveness and thus has the power to restore a broken relationship. Here we showed that apology from the offender not only reduces the victim’s propensity to react aggressively but also alters the victim’s implicit attitude and neural responses toward the offender. We adopted an interpersonal competitive game which consisted of two phases. In the first, “passive” phase, participants were punished by high or low pain stimulation chosen by the opponents when losing a trial. During the break, participants received a note from each of the opponents, one apologizing and the other not. The second, “active” phase, involved a change of roles where participants could punish the two opponents after winning. Experiment 1 included an Implicit Association Test (IAT) in between the reception of notes and the second phase. Experiment 2 recorded participants’ brain potentials in the second phase. We found that participants reacted less aggressively toward the apologizing opponent than the non-apologizing opponent in the active phase. Moreover, female, but not male, participants responded faster in the IAT when positive and negative words were associated with the apologizing and the non-apologizing opponents, respectively, suggesting that female participants had enhanced implicit attitude toward the apologizing opponent. Furthermore, the late positive potential (LPP), a component in brain potentials associated with affective/motivational reactions, was larger when viewing the portrait of the apologizing than the non-apologizing opponent when participants subsequently selected low punishment. Additionally, the LPP elicited by the apologizing opponents’ portrait was larger in the female than in the male participants. These findings confirm the apology’s role in reducing reactive aggression and further reveal that this forgiveness process engages, at least in female, an enhancement of the victim’s implicit attitude and a prosocial motivational change toward the offender.

## Introduction

Interpersonal conflicts are ubiquitous in our social life. A natural self-defense mechanism in many social species is the desire for revenge, that is, to react aggressively toward the offender. However, reacting in accordance with the “eye-for-an-eye” principle also carries adverse effects and ultimately leads to the breakdown of interpersonal relationships (Carlsmith et al., 2008; Rand et al., 2009; Wu et al., 2009). In fact, humans possess an important virtue, which is the ability to forgive. Social psychologists define forgiveness as a set of changes whereby one feels decreased negative emotions toward the offender, reduced motivation to retaliate or punish, and an increase in will to continue the relationship despite the offender’s hurtful actions (McCullough et al., 1997; Worthington, 2006). In other words, forgiveness acts to rebuild the damaged relationship. Yet, in real life, unconditional forgiveness as a pure gift is not easily affordable (Griswold, 2007; Hughes, 2015). The key process to avoid revenge and overcome the negative feelings of resentment in the victim is for the offender to give an apology. The offender must acknowledge his/her responsibility and express remorse (Lazare, 2004), demonstrate that he/she is a trustful person, and that both parties share the same moral values. In these terms, apology meets the conditions required for forgiveness, and constitutes a crucial remedy for interpersonal conflict.

The positive impact of apology on reconciliation and forgiveness is well established in social psychological research (Darby and Schlenker, 1982; McCullough et al., 1997; Exline and Baumeister, 2000; De Cremer et al., 2011). Factors mediating the influence of apology have also been extensively studied, including the severity of the offense and level of responsibility (Schlenker and Darby, 1981; Bennett and Earwaker, 1994), the intention to offend (Struthers et al., 2008), the level of elaboration of apology (Darby and Schlenker, 1982; Kirchhoff et al., 2012) and the time lapse between offense and apology (Frantz and Bennigson, 2005). There is evidence that apology from an offender influences the victim at the affective, cognitive, and behavioral levels. At the cognitive level, apology affects victims’ perception of the offender such that they make more positive attributions about the one who apologizes (Darby and Schlenker, 1982; Ohbuchi et al., 1989). At the affective level, apology can help reduce the victim’s negative emotions such as anger (Ohbuchi et al., 1989; Kirchhoff et al., 2012) and increase empathy toward the offender (McCullough et al., 1997). At the behavioral level, the recipient of apology is more likely to refrain from retaliatory and aggressive behavior (Gold and Weiner, 2000; Strang et al., 2014).

While much is known about the consequences of apology, little is known about the implicit and neural impact substantiating those outcomes. As far as we know, there is only one recent neuroimaging study investigating the neural correlates of receiving an apology and actively forgiving offense in a two-person interactive game (Strang et al., 2014). In this study, participants were asked if they wanted to forgive another player when the latter made a choice with negative consequences for them. Before the decision to forgive, the participants either received an apology or not from the other player. The authors found that participants forgave more often after an apology message and that receiving an apology yielded activation in empathy-related brain regions. However, several features in their design may have rendered their interpretations ambiguous. First, as the offenses and the decisions not to forgive involved losing money for the participants and/or the offender, other psychological factors such as fairness consideration, strategic thought, and self-interest might have influenced the behavior. Moreover, since the participants were explicitly asked at each trial if they forgave the player or not, they could be forced to abide by the social norm (i.e., forgiving transgressors if they repent) and falsely express their forgiveness of the apologizing offenders. To avoid these potential pitfalls, here we aimed to utilize more implicit measures to examine the victim’s reactions to apology that are otherwise not visible in explicit measures and behavior. With a more naturalistic setting, we combined behavioral and electrophysiological (event-related potential, ERP) measures and investigated, at the cognitive, affective, and behavioral levels, the direct, implicit transformations elicited by apology. Note, we used the ERP technique to measure brain responses to forgiveness as its impact unfolds over time, rather than brain regions involved in forgiveness, as Strang et al. (2014) did.

**FIGURE 1:**
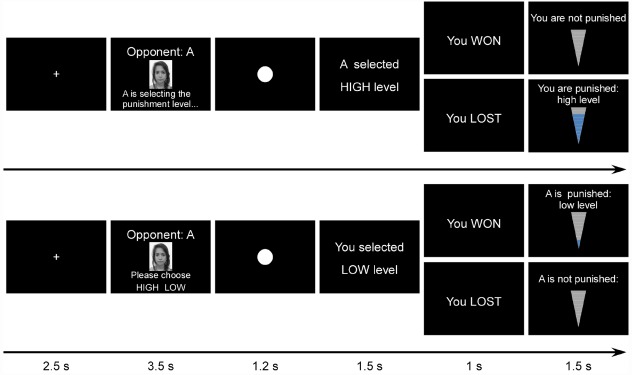
**Task display and timing of Experiment 1.** Top panel: passive phase. Bottom panel: active phase.

In two experiments, we adopted a modified version of the Taylor Aggression Paradigm (TAP; Taylor, 1967) divided into two phases (Figure 1). The first, “passive” phase was designed so that the participant was passively punished (received painful stimulation) by two different opponents each time he/she lost a trial (i.e., responded slower than the opponent) in a reaction-time competition task. In this phase, the aggressiveness of the opponents was predetermined such that they systematically chose more high than low intensity punishment for the participant. After the first phase, one opponent sent an apologizing message and the other a non-apologizing message to the participant. In the second, “active” phase, the roles of the participant and the two opponents were exchanged: the participant became an active partner and had the right to punish the opponents when they lost a trial. Our design allowed us to measure the changes induced by apology at the three distinct levels mentioned above. Compared with existing studies we attempted to measure implicit reactions in addition to participants’ explicit self-reports. First, at the behavioral level, as an index of the retaliation/forgiveness behavior we measured the severity of the reactive punishment administrated by the participant to each of the opponents during the second phase (Experiments 1 and 2). Second, at the cognitive level, in order to measure their attitude toward the apologizing and non-apologizing opponent, we administrated an Implicit Association Test (IAT; Greenwald et al., 1998) right after the participant received the apologizing and non-apologizing messages (Experiment 1). Third, at the affective/motivational level, we recorded and analyzed ERPs of the participants (Experiment 2).

We analyzed EEG responses during the decision phase and the outcome phase in Experiment 2. For the decision phase (when participants were deciding the intensity of punishment), we focused on N2 and the late positive potential (LPP). The former component, a negative deflection of brain potential peaking around 200 ms after stimulus, has been associated with aggressiveness in a previous study using TAP (Krämer et al., 2008). If apology reduces aggressiveness, we hypothesized that N2 should show a larger amplitude for the non-apologizing opponent than for the one who apologized. The LPP is a sustained positive component distributed mainly in the posterior part of the brain, which has been consistently associated with the processing of emotional stimuli, irrespective of the valence of the affective arousal (Schupp et al., 2000; Sabatinelli et al., 2007). The pattern of LPP could help us gain insight into the effect of apology on the affective/motivational reaction underlying the decision to punish the offender. For the outcome stage, where participants learned if they won or lost the trial, we focused on the feedback-related negativity (FRN) and P300. As these components are sensitive to outcome evaluation (e.g., Gehring and Willoughby, 2002; Hajcak et al., 2005), we sought to investigate whether apology influences the affective/motivational reaction to win or loss trials. Given that FRN is usually more pronounced for negative feedbacks, it is possible that, if participants have stronger retaliation motivation toward the non-apologizing opponent, losing a trial against the non-apologizing opponent (i.e., who would then not be punished in that trial) would elicit a larger amplitude than losing a trial against the apologizing opponent. In contrast, the P300 response has been found to be stronger for positive than negative rewards; because apology reduces the motivation to punish, winning against the opponent who did not apologize (which leads to punishment for the opponent) would be felt as more positive and rewarding than winning against the apologizing opponent.

We believe that these different measures are conceptually related and can provide insights from different angles into the psychological processes motivating a victim to forgive. Given that gender plays a significant role in social and affective processes, especially in dealing with aggressive behavior (Bettencourt and Miller, 1996), we were also interested in whether gender could moderate the effect of apology on interpersonal forgiveness.

## Experiment 1: Behavioral Experiment

### Methods

#### Participants

Thirty-six graduate and undergraduate students (aged between 19 and 25 years, 17 males; none from psychology or related disciplines) took part in this experiment. All the participants were right handed, with normal or corrected-to-normal vision. None of them had a history of neurological, psychiatric, or cognitive disorders. All the participants were informed of the properties of the pain stimulation in detail during the recruitment and before the experiment began. Informed written consent was obtained from each participant before the test. This study was carried out in accordance with the Declaration of Helsinki and was approved by the Ethics Committee of the Department of Psychology, Peking University.

#### Tasks

***The modified Taylor Aggression Paradigm***

The TAP is a frequently used method to elicit and measure aggressive behavior in a laboratory setting. In TAP, participants are led to believe that they are playing a competitive reaction time task against one or more opponents. In reality, both the outcome of the reaction time and the opponents’ behavior are under control of the experimenter. In the classical TAP, the winner of the task from each trial gets to punish the loser with an aversive stimulus of variable intensity. We modified the classical TAP so that the participant played the game in two phases. During the first (passive) phase, the participant could only be punished (to elicit aggressive retaliation motivation), in the second (active) phase he was the one able to punish the opponents (to measure aggressive reactive behavior). The experimental conditions were manipulated between the first and the second phase, i.e., one opponent wrote an apologizing note, and the other one did not apologize in his note.

The punishment was moderately painful electric stimulations. The use of electric shock has been used in a number of studies investigating social emotions (e.g., Crockett et al., 2014, 2015; Winston et al., 2014). It has the benefits of eliciting more primitive instincts and more intensive emotional arousals than monetary loss (which is widely adopted as a way of interpersonal transgression). It is presumably less vulnerable to inter-individual variations. An intra-epidermal needle electrode was attached to the left wrist of the participant for cutaneous electrical stimulation (Inui et al., 2002). Great care was taken to ensure that no permanent damage could occur. The participants were informed, at the time of recruitment and before the experiment, that the stimulation would not produce any irreversible effect. Two participant-specific pain intensities were calibrated such that the high intensity stimulation was rated as 8 and the low intensity was rated as 3 on a 0–10 scale (0, no sensation; 10, unbearably painful).

***The Implicit Association Test***

We employed an IAT (Greenwald et al., 1998) to measure the participant’s implicit attitude toward the apologizing and non-apologizing opponents. Compared to explicit measures, such as self-report and behavioral punishment, IAT has the strength to assess unconscious and automatic responses to social and affective stimuli, largely unaffected by the influence of reputation, social desirability, and self-image (cf. Phelps et al., 2000). For our study, the participant had to associate belongings from the apologizing and non-apologizing opponents (memorized before the task) with either negative or positive attributive words. This modified version of IAT was used in a number of previous studies (e.g., Huang et al., 2009; Wu et al., 2013). We hypothesized that participants would respond faster to the apologizing opponent with positive attributive words and to the non-apologizing opponent with negative attributive words (congruent trials), and slower for the non-apologizing opponent’s belonging with positive attributes and apologizing opponent with negative words (incongruent trials).

#### Design and Procedure

Upon arrival, each participant was told that he/she would later play an interactive game together with two opponents already in another room, via intranet. We first measured the pain threshold of the participant and determined the two critical pain intensities for each participant. The low intensity corresponded to the participant’s self-report of 3 and the high intensity corresponded to 8 on a scale ranging from 0 to 10. Then each participant was told that the experiment was divided into two parts: first a passive phase during which the participant would be passively punished by the two opponents each time he/she lost a trial. Then an active phase where the participant could actively punish the opponents when they lost. The participant was made to believe that the opponents did not know about the role switching until the second phase.

During the whole experiment, the participant did not meet the two opponents (confederates); the identity of the two opponents was given by his/her (facial) portrait and the label A and B through the intranet. The two opponents were of the same sex as the participant and the associations between portraits and apologizing/non-apologizing were counterbalanced over participants.

***Phase 1: passive phase***

At the beginning of each trial (Figure 1, top panel), the computer presented the identity of the opponent (the portrait and the label A or B), indicating against whom the participant was playing for this trial and that this opponent (the active player) was selecting the intensity of the punishment (high or low). Then the reaction time task required the players to press a button (“space key”) as fast as possible when a white dot appeared in the center of screen. The punishment intensity chosen by the opponent was subsequently presented on the screen. After that, the outcome of the reaction-time game was displayed. If the opponent won the trial (i.e., responded faster than the participant), the participant would receive the punishment with the intensity chosen by the opponent at the beginning; if the opponent lost the trial, the participant would not be punished. In fact, the outcome of each trial was predetermined by the experimenters.

The participant played as the passive player for a total of 64 trials. For each trial, one of the two opponents (A or B) was randomly selected by the computer to interact with the participant in that trial. A and B opponents were each selected for 32 trials. The probability of winning a trial was 50% for both A and B and the proportion of high intensity punishment chosen by A and B was 75% (24 trials) in total. All the trials were pseudorandomized and the condition with the same punishment intensity would not appear more than three consecutive times.

***Apology manipulation during the break time***

After the first passive phase, participants and the opponents had a break time during which the participant received one message from each opponent, which was passed on by the experimenter (the participant did not meet the opponent directly throughout the experiment). Specifically, one opponent apologized to the participant while the other did not. The message from the apologizing opponent was: “Sorry, the punishments I gave you were a bit high, I will modify my choices for the next part. Sorry again for the harm I caused to you.” The message from the non-apologizing opponent was: “I find this game rather exciting, I guess the electrical stimulation does not hurt that much, so I chose some higher intensity.” The opponent labels (A or B) and the apologizing/non-apologizing messages were counterbalanced over participants.

After the participant read the messages, he/she completed a number of subjective ratings. On a 7-point scale, he/she answered his/her level of unhappiness, anger, willingness to forgive, willingness to punish, willingness to be a friend, and impression for the two opponents respectively. For the “impression” item, 1 means “very bad,” and 7 means “very good.” For the other items, 1 means “not at all,” and 7 means “extremely strong.”

***Implicit Association Test***

Right after the completion of the subjective ratings, the IAT began. Each participant first had to take 2 min to memorize and associate a number of objects/belongings (target stimuli) to their owners (i.e., the opponents, A and B). Then, the participant performed seven IAT blocks (Table 1) in which he/she was instructed to respond to target stimuli and/or attributive words as correctly and quickly as possible. The first two blocks were training blocks. In Block 1, the participant pressed one key (F or J on the keyboard) when A’s belongings were presented, and the other key for B’s belongings. In Block 2, he/she pressed one key for positive words and the other for negative words. In Block 3 and Block 4, the participant pressed one key for A’s belongings or positive words, and pressed another key for B’s belongings or negative words. Block 3 served as a training block, familiarizing the participant with the key codes, and Block 4 served as a testing block. In Block 5, the key code for the belongings switched and the participant had to respond to belongings only, as in Block 1. It should be noted that the key code for the attributive words remained the same throughout the whole IAT experiment. Block 6 and Block 7 were similar to Block 3 and Block 4, except that the key code for the belongings switched. Given that we hypothesized that the participant has positive attitude toward the apologizing opponent and negative attitude toward the non-apologizing opponent, we defined the congruent block as the testing block in which the apologizing opponent’s belongings and positive attributive words shared the same key, and defined the incongruent block as the testing block in which the apologizing opponent’s belongings and negative attributive words shared the same key. The order of congruent and incongruent blocks was counterbalanced across participants. A red “X” appeared at the center of the screen after every incorrect response, i.e., when the participant responded with the wrong key.

**TABLE 1:** **Procedure of the Implicit Association Test**.

**Block**	**Task (number of trials)**	**Corresponding key**	
		**Left key (F)**	**Right key (J)**
i	Target stimuli reaction (24)	A belongings	B belongings
ii	Attributive words reaction (24)	Positive words	Negative words
iii	Initial association task (24)	A belongings/positive words	B belongings/negative words
**iv**	**Initial association task (48)**	**A belongings/positive words**	**B belongings/pegative words**
v	Reversed target stimuli reaction (24)	B belongings	A belongings
vi	Reversed association task (24)	B belongings/positive words	A belongings/negative words
**vii**	**Reversed association task (48)**	**B belongings/positive words**	**A belongings/negative words**

Blocks in bold are testing blocks.

We analyzed the reaction times for the fourth and seventh blocks (i.e., the testing blocks) in the IAT experiment. Note again, for half of the participants, the fourth block was the congruent block, in which the apologizing opponent’s belongings and positive words shared the same response key, and the seventh block was the incongruent block, in which the apologizing opponent’s belongings and negative words shared the same response key. For the other half, the fourth block was the incongruent block, and the seventh block was the congruent block. The potential influence of test order was therefore counter-balanced in this procedure.

One group of target stimuli (belongings) contained “figurine,” “ruler,” and “candy” (in words), and the other group, “chocolate,” “cup,” and “pen” (in words). Positive attributive words included “sunshine,” “luck,” “love,” “happiness,” “joy,” “fun,” “festival,” and “friendship”; negative attributive words included “disease,” “death,” “murder,” “accident,” “poison,” “war,” “tragedy,” and “vomit.” Inquisit four software was employed to present stimuli in IAT. The two groups of target stimuli were assigned to the opponents A and B, respectively. This assignment was counterbalanced over participants.

***Phase 2: active phase***

For the second, active phase, the participant and the two opponents exchanged roles. The participant became the active player while the two opponents became the passive players. The participant was told at the beginning of the experiment that only he/she knew that the roles would be exchanged, while the opponents did not learn about this manipulation until the beginning of the second phase. This information was given to eliminate the participant’s potential concern about a strategic apology (i.e., giving an apology just to avoid undergoing the revenge of the participant and be punished in the next part). In other words, the participant was made to believe that the opponent apologized sincerely, without knowing that he/she would be punished later. At the beginning of each trial (Figure 1, bottom panel), the portrait of the opponent and the corresponding label was presented on the screen and the participant had to choose the pain intensity for this opponent. The participant pressed two buttons to choose from two intensity levels. The key codes were counterbalanced over participants. The rest of the trial sequence was similar to the passive phase: the participant had to press the space key as the white dot appeared on the screen, then the participant was presented with the punishment intensity he selected earlier in the trial, followed by the outcome of the reaction time task. At the end of the trial, the outcome of the reaction-time game was displayed. In contrast to the passive phase, if the participant won the trial, the opponent would receive the punishment with the intensity chosen by the participant at the beginning; if the participant lost the trial, the opponent would not be punished. All trials were pseudorandomized such that the same condition would not appear three or more consecutive times. Similar to the passive phase, the active phase consisted of 64 trials. The two opponents interacted with the participant respectively for 32 trials, whose performance was in fact controlled by our program. The proportion of winning trials was 50% for both opponents. After this second phase, the participants were paid and thanked. No participants expressed suspicion of the experiment manipulation.

***Measurements***

The intensity of punishments that the participant selected for the two opponents in the second phase of the TAP was used as an index for the retaliation/forgiveness behavior. For the IAT (implicit attitude), we analyzed the reaction times of congruent and incongruent trials. Steps for the analysis followed the procedure implemented in previous research (i.e., Wu et al., 2013). (1) We removed one participant whose error rate was over 20%, leaving 35 participants for further data analysis. (2) We excluded all the error trials from the analysis of reaction time, i.e., when the participant answered with the wrong response key (average error rate: 5.8%). (3) From the remaining trials, those in which participants did not respond within 3 s and trials in which the reaction times exceeded three standard deviations from the mean in each experimental condition were excluded from the data analysis (0.18% of the trials). Thus, in total, less than 6% of the total trials were excluded.

### Results

#### Subjective Ratings

Ratings on the six items after receiving the messages of the two opponents did not show any significant difference between the two opponents (Table 2). There was no gender difference either.

**TABLE 2:** **Subjective ratings for apologizing/non-apologizing opponents in Experiment 1**.

	**Apologizing opponent (Mean ± SD)**	**Non-apologizing opponent (Mean ± SD)**	***t*-value (*n* = 36)**	***p*-value**
Unhappy	2.08 ± 1.32	2.39 ± 1.62	–1.43	0.160
Anger	1.71 ± 0.98	1.89 ± 1.27	–1.27	0.213
Forgiveness	5.76 ± 1.64	5.84 ± 1.50	–0.27	0.791
Willingness to punish	4.13 ± 1.30	4.00 ± 1.27	0.68	0.500
Willingness to be friend	5.61 ± 1.29	5.37 ± 1.36	1.10	0.277
Impression	5.74 ± 1.13	5.55 ± 1.29	1.16	0.255

After receiving the opponents’ messages but before the active phase, the participant rated on a 7-point scale about the degree to which he/she felt on the above dimensions. For the “impression” item, 1 means “very bad”, and 7 means “very good”. For the other items, 1 means “not at all,” and 7 means “extremely strong”.

#### IAT Reaction Time

**FIGURE 2:**
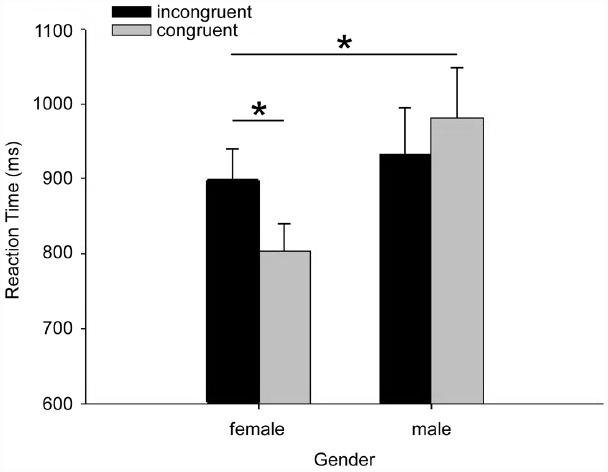
**IAT reaction time (Error bars represent standard deviation of the mean value).** Congruent: apologizing opponent belongings-positive words/non-apologizing belongings-negative words; incongruent: apologizing opponent belongings-negative words/non-apologizing belongings-positive words). Significance indicators: *p < 0.05.

To examine the impact of apology on the implicit attitude of the victim toward the offenders, we used an IAT construct (Greenwald et al., 1998) to reflect the implicit attitude (positive or negative) toward the apologizing or non-apologizing opponent. Shorter response times in the congruent block and longer response times in the incongruent block indicated stronger association between the apologizing opponent (relative to non-apologizing opponent) and positive concept. The association with positive/negative concept was interpreted as reflecting the participant’s implicit attitude toward the target objects. Here, we carried out a two-way ANOVA with congruency as a within-participant factor and gender as a between-participant factor. The interaction was significant, *F*(1,33) = 4.76, *p* = 0.036. Pair-wise comparisons were carried out separately for each gender (Figure 2). We found that the reaction times for the female participants in the congruent condition (*M* = 786 ms, SD = 132) were significantly faster than those in the incongruent condition (*M* = 885 ms, SD = 171), *F*(1,33) = 5.7, *p* = 0.022, while there was no significant difference between the two conditions for male participants (congruent: *M* = 936 ms, SD = 299; incongruent: *M* = 906 ms, SD = 221). The main effects of congruency, *F*(1,33) = 1.34, *p* = 0.25, and gender, *F*(1,33) = 2.43, *p* = 0.13, were not significant. For error rate, no significant difference was found between genders, *F*(1,30) = 1.16, *p* = 0.29, or between congruent and incongruent conditions, *F*(1,30) = 0.48, *p* = 0.43. However, the interaction between gender and congruency was significant, *F*(1,30) = 4.3, *p* = 0.047. Specifically, pairwise comparisons revealed that male participants’ error rates were higher in the congruent condition (*M* = 4.73, SD = 6.47) than in the incongruent condition (*M* = 2.73, SD = 2.89), *F*(1,30) = 3.6, *p* = 0.067; whereas female participants’ error rates were higher for the incongruent condition (*M* = 2.94, SD = 3.51) than for the congruent condition (*M* = 1.94, SD = 1.48), although this effect did not reach statistical significance, *F*(1,30) = 1.02, *p* = 0.32.

#### Reactive Punishment

To examine the effect of apology on the reactive aggressive behavior, we examined the punishment behavior toward the two types of opponents. For the second, “active” phase, the dependent variable was the proportion of high intensity punishment chosen by the participant. We carried out an ANOVA with opponent (apologizing vs. non-apologizing) as a within-participant factor and gender of the participant as a between-participant factor. The main effect of opponent was significant, *F*(1,34) = 5.99, *p* = 0.020. Participants’ choices of high punishments for the apologizing opponents (*M* = 0.43, SD = 0.18) were significantly lower than those chosen for the non-apologizing opponents (*M* = 0.47, SD = 0.18). The main effect of gender was not significant, *F*(1,34) = 0.54, *p* = 0.47. The interaction between the two factors was not significant either, *F*(1,34) = 0.02, *p* = 0.89. We tested the correlation between the apology effect on behavior (the difference between punishment for non-apologizing and apologizing opponent) and the congruency effect in IAT (the difference between RT in incongruent and congruent trials). The correlation was not significant, *r* = 0.165, *p* = 0.34.

### Discussion

In line with the philosophical and psychological definitions of forgiveness, the behavioral data showed that participants reduced the proportion of high intensity punishments for the apologizing opponent relative to the non-apologizing opponent. Moreover, the IAT results, measured before the active phase, revealed that female participants responded significantly faster in the congruent block than in the incongruent block, suggesting that they had a more positive attitude toward the apologizing than the non-apologizing opponent. However, male participants did not show significant difference in implicit attitude toward the two opponents. This null effect will be discussed later on. In general, findings from this experiment suggest that after an interpersonal transgression, the forgiveness process is facilitated by apology. Specifically, apology reduces exterior reactive aggression behavior for both male and female, and induces changes in the implicit attitude toward the apologizing offender, at least for females. Finally, the results indicated no significant correlation between IAT and behavioral punishment.

## Experiment 2: EEG Experiment

### Methods

#### Participants

We recruited 26 graduate and undergraduate students (10 males, aged between 19 and 24; none from psychology or related disciplines) for this experiment. None of them had participated in Experiment 1.

#### Tasks

The experiment was similar to Experiment 1: the participant was the passive player for the first phase and then the active player in the second phase. In this Experiment, EEG data were collected during the second phase. Moreover, to avoid potential influences on brain activity, we did not administrate the IAT after the reception of the apologizing and non-apologizing messages.

#### Procedure

The experimental procedure was essentially the same as in Experiment 1, except that there was no IAT between the two phases.

In the first phase, we increased the number of trials from 64 to 100 and raised the proportion of high intensity punishments selected by the opponents from 75% to 80%. These changes were aimed to enhance the magnitude of the offense and the reactive aggression in the participants.

**FIGURE 3:**
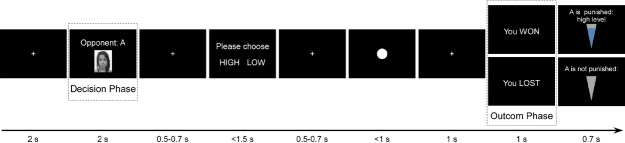
**Task display and timing of Experiment 2.** Active phase, when the participant selects high level punishment. The critical events for EEG analysis are marked with dash line.

In between the first and second phases, after the participant read the two messages from opponents, he/she carried out the subjective ratings (the same as in Experiment 1). Then the second phase began with the participant being the active player. In this phase EEG data were collected (Figure 3).

For the second phase, the number of trials increased to 160; the participant played 80 trials with each opponent, with the winning trials kept at 50%, similar to the first experiment. The larger number of trials was required by the EEG recording and analysis.

Each trial had a decision phase, during which the (face) portrait and label (A or B) of the opponent were presented, informing the participant that he/she would have to choose the punishment intensity for this opponent. After this decision phase was the reaction time competition task. Then came the outcome (feedback) phase, during which the result of the reaction time game was displayed on the screen (Figure 3). We analyzed the neural activity for the decision and the outcome phases, respectively.

At the end of Experiment 2, we administrated a manipulation check: the two opponents’ portraits were presented to the participant on a white sheet. The participant had to write the letter (A or B) corresponding to their labels. Then the participant recalled the opponents’ messages between the passive and active phases and indicated which one had expressed apology in a forced-choice question (“Who has expressed apology to you, A or B?”). No participants expressed suspicion of the experimental manipulation.

#### EEG Recording

The EEG data were recorded using a 64-channel Brain Products system (online pass band: 0.061–100 Hz, sampling rate: 1000 Hz), connected to a standard EEG cap according to the international 10–20 system. The electrodes were localized at the frontal area (FP1, FP2, AF7, AF3, AF4, AF8, F7, F5, F3, F1, Fz, F2, F4, F6, and F8), central area (C5, C3, C1, Cz, C2, C4, and C6), parietal area (P7, P5, P3, P1, Pz, P2, P4, P6, and P8), temporal area (FT7, FT8, T7, T8, TP7, and TP8), occipital area (O1, Oz, and O2), fronto-central area (FC5, FC3, FC1, FCz, FC2, FC4, and FC6), centro-parietal area (CP5, CP3, CP1, CPz, CP2, CP4, and CP6), and parieto-occipital area (PO7, PO5, PO3, POz, PO4, and PO8). The nose was used as online reference channel, and all channels impedances were kept lower than 10 kΩ. To monitor ocular movements and eye blinks, electro-oculographic (EOG) signals were simultaneously recorded from four surface electrodes, one pair placed over the higher and lower eyelid of left eye, the other pair placed lateral to the outer canthus of the each eye.

#### EEG Data Analysis

Standard procedure for data analysis was employed for the analysis of ERP data (Luck, 2005, Chap. 4). We used Analyzer 2.0 software to analyze the EEG recordings. EEG data were re-referenced offline to the mean of the left and right mastoids. The EEG data contaminated by eye-blinks and movements were corrected using an independent component analysis (ICA) algorithm as implemented in the software. For both the decision phase and the outcome phase, EEG epochs were extracted using a time window of 1000 ms (200 ms pre-stimulus and 800 ms post-stimulus), and baseline corrected using the pre-stimulus time interval. All trials in which EEG voltages exceeded a threshold of ±85 μV during recording were excluded. The EEG data were low-pass filtered below 30 Hz.

***Decision phase***

From the grand average ERPs across all the participants in the decision phase, N2 and the LPP were analyzed.

**FIGURE 4:**
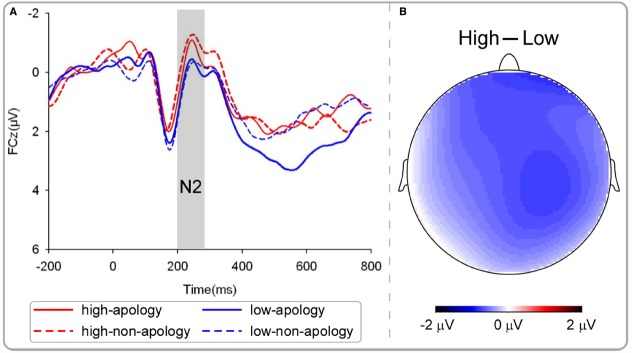
**EEG results of the decision phase: N2. (A)** Grand average ERP. **(B)** Topography of high—low punishment condition.

N2, a fronto-centrally distributed negativity around 200–300 ms post-onset, was defined as the mean amplitudes in the time window of 200–280 ms. N2 has been associated with aggressiveness in a previous study (Krämer et al., 2008). EEG data from three participants were excluded due to excessive artifact contaminations within this time window (leaving 23 participant for analysis). For these participants, the number of trials for at least one condition was less than 10 trials (about 30% of the total number of trials in that condition) after artifact rejection. For the simplicity of statistical analysis, we focused on the FCz electrode. We performed a three-way ANOVA with opponent (apologizing vs. non-apologizing) and the punishment intensity that the participant subsequent chosen (high vs. low) as the within-participant factors, and participants’ gender as the between-participant factor. Effects over the whole scalp are illustrated with the topographic map (Figure 4).

**FIGURE 5:**
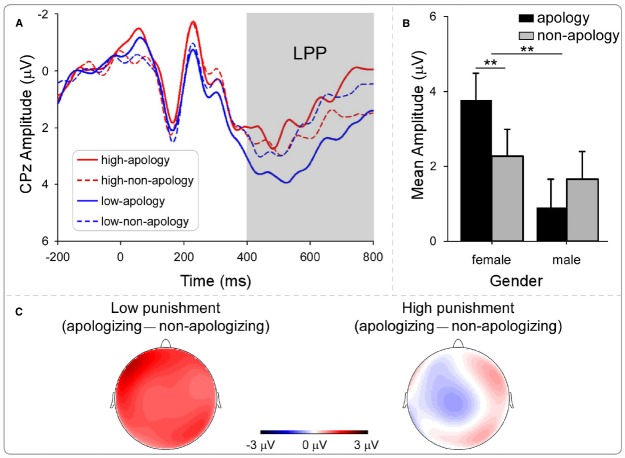
**EEG results of the decision phase: LPP. (A)** The grand average ERP in the decision phase condition of LPP. **(B)** LPP mean amplitude as a function of opponent and participants’ gender. **(C)** Topography of “apologizing—non-apologizing” in high and low punishment. Significance indicators: **p < 0.001.

Late positive potential, a component strongly modulated by the emotional intensity of a stimulus (Schupp et al., 2000; Sabatinelli et al., 2007), was defined as the mean amplitudes in the time window of 400–800 ms. EEG data from the same three participants were excluded due to excessive artifact contaminations within this time window. From the grand average ERPs across all the participants in the decision phase, we chose five electrodes along the midline (Fz, FCz, Cz, CPz, and Pz) to represent the LPP component. For statistical analysis of the magnitude of LPP, we carried out a four-way ANOVA with opponent (apologizing or non-apologizing), punishment intensity (high and low), and electrode position (five levels: Fz, FCz, Cz, CPz, and Pz) as the within-participant factors and the participant’s gender as the between-participant factor. Again, effects over the whole scalp are illustrated with the topographic map (Figure 5). The rationale for the selection of the electrodes for N2 and LPP was that the grand average ERPs showed the strongest effects on the corresponding electrodes for these components and that the electrodes are typically reported for these components in the literature (see, for example, Smillie et al., 2011; Moser et al., 2006, for similar methods of electrodes selection). PASW 20 software was used in the statistical analyses. The Greenhouse–Geisser correction for violation of the ANOVA assumption of sphericity was applied where appropriate. Bonferroni correction was used for multiple comparisons.

***Outcome phase***

We analyzed ERPs during the outcome phase to see if apology had an effect on the affective/motivational evaluation of win or loss trials. For the grand average ERPs over all the participants in the outcome phase, the FRN and P300 were analyzed. EEG data from four participants were excluded due to excessive artifact contaminations within the time windows, leaving 22 participants for data analysis.

**FIGURE 6:**
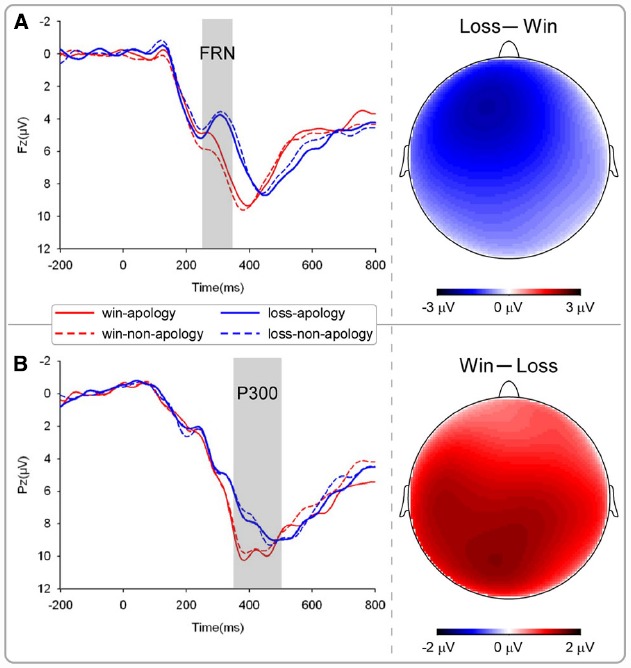
**EEG results in the outcome phase.** The grand average ERPs of **(A)** FRN and **(B)** P300.

Feedback-related negativity is a negative deflection at fronto-central recording sites; we defined it as the mean amplitudes in the time window of 250–300 ms. The number of trials for at least one condition was less than 20 trials (about 50% of the total number of trials in that condition) after artifact rejection. For the simplicity of statistical analysis, we focused on the Fz electrode. We performed a three-way ANOVA with opponent (apologizing vs. non-apologizing) and outcome (win vs. loss) as the within-participant factors, and participants’ gender as the between-participant factor. Effects over the whole scalp are illustrated with the topographic map (Figure 6).

P300 is the most positive peak between 200 and 600 ms post-onset of feedback; here it was defined as the mean amplitudes in the time window of 350–500 ms. For statistical analysis, we focused on the Pz electrode. We performed a three-way ANOVA with opponent (apologizing vs. non-apologizing) and outcome valence (win vs. loss) as the within-participant factors, and participants’ gender as the between-participant factor. Again, effects over the whole scalp are illustrated with the topographic map (Figure 6).

### Results

#### Manipulation Checks and Subjective Ratings

In the post-experiment manipulation check, all of our participants correctly assigned the labels to the corresponding opponents and accurately recalled who had apologized. We can thus confirm that our manipulation was successful. For the subjective ratings, we carried out a two-way ANOVA for each item with apology as the within-participant factor and gender as the between-participant factor (Table 3). There were no significant gender differences. There was only a significant main effect of the opponent for the willingness to punish, *F*(1,24) = 6.25, *p* = 0.02. Specifically, the willingness to punish was lower for the apologizing opponent (*M* = 4.12, SD = 0.77) than the non-apologizing opponent (*M* = 4.65, SD = 0.85).

**TABLE 3:** **Subjective ratings for apologizing/non-apologizing opponents in Experiment 2**.

	**Apologizing opponent (Mean ± SD)**	**Non-apologizing opponent (Mean ± SD)**	***t*-value (*n* = 26)**	***p*-value**
Unhappy	2.62 ± 1.39	2.73 ± 1.54	–0.36	0.722
Anger	2.12 ± 1.40	2.35 ± 1.29	–0.84	0.407
Forgiveness	5.85 ± 1.35	5.46 ± 1.63	1.10	0.284
Willingness to punish	4.12 ± 0.77	4.65 ± 0.85	–2.67	0.013*
Willingness to be friend	5.19 ± 1.30	4.88 ± 1.56	0.96	0.349
Impression	4.85 ± 1.26	4.65 ± 1.38	0.71	0.486

After receiving the opponents’ messages but before the active phase, the participant rated on a 7-point scale about the degree to which he/she felt on the above dimensions. For the “impression” item, 1 means “very bad,” and 7 means “very good.” For the other items, 1 means “not at all,” and 7 means “extremely strong.” *p < 0.05.

#### Reactive Punishment

The dependent variable for the active phase was the proportion of high punishment chosen by the participant. We carried out a repeated-measure ANOVA with the opponent (apologizing vs. non-apologizing) as the within-participant factor and the gender of the participants as the between-participant factor. The main effect of opponent was significant, *F*(1,24) = 8.052, *p* = 0.009. The proportion of high punishments chosen for the apologizing opponent (*M* = 0.48, SD = 0.14) was significantly lower than for the non-apologizing opponent (*M* = 0.56, SD = 0.16). The main effect of gender was not significant, *F*(1,24) = 0.34, *p* = 0.56, nor was the interaction between gender and apology, *F*(1,24) = 3.107, *p* = 0.091.

#### EEG Results

To further examine the impact of apology on the neural and psychological processes associated with forgiveness, we analyzed the neural response of participants when they were indicating for which opponent they would chose the punishment intensity (the decision phase) and when they were presented with the outcome of the reaction-time frame (outcome phase).

***Decision phase***

*N2*. In a previous study using TAP (Krämer et al., 2008), larger N2 amplitudes have been observed in high trait aggressive participants in response to high provocation, relative to low provocation. Given that N2 is interpreted as reflecting the conflict between aggressive impulse and cognitive control, we hypothesized that the amplitude would be larger (more negative) when selecting punishment intensity for the non-apologizing opponent relative to the apologizing opponent. We carried out a three-way ANOVA with opponent (apologizing vs. non-apologizing) and punishment intensity that the participant subsequently chose (high vs. low) as within-participants factors, and gender as a between-participant factor. Inconsistent with our hypothesis, the only significant effect revealed by this analysis was a significant main effect of punishment intensity, *F*(1,21) = 8.96, *p* = 0.007 (Figure 4). The mean amplitude of high punishment (*M* = –0.57 μV, SD = 2.52) was significantly more negative than that of low punishment (*M* = 0.18 μV, SD = 2.65).

*LPP*. Previous studies have shown that increased positive amplitudes reflect enhanced motivated attention to emotional stimuli (Hajcak and Olvet, 2008; Van Hooff et al., 2011). Therefore, if LPP amplitude was stronger for the non-apologizing opponent, this would suggest that the stronger emotional salience of this opponent motivated the participant to inflict higher punishments. If LPP amplitude was larger for the apologizing opponent, it would suggest that the motivation elicited by apology leads the participant to behave more prosocially toward the apologizing opponent rather than behave more aggressively toward the opponent who did not apologize. The grand average LPPs at the CPz electrode are shown in Figure 5A. We carried out a four-way repeated-measures ANOVA on the LPP mean amplitudes, with apology (apologizing vs. non-apologizing), punishment intensity (high vs. low), and electrode position (Fz, FCz, Cz, CPz, and Pz) as within-participant factors, and the participant’s gender as a between-participant factor. The main effect of electrode position was not significant, *F*(4,19) = 1.571, *p* = 0.216, neither was any interaction involving the electrode. Therefore, we collapsed the five electrodes position and carried out a three-way ANOVA with the three factors left. The three-way interaction was not significant, *F*(1,21) = 0.518, *p* = 0.480, but we found two significant two-way interactions. First, the interaction between punishment intensity and opponent was significant, *F*(1,21) = 4.232, *p* = 0.052 (Figures 5A,C). Pair-wise comparison showed that when participants chose low punishment, the amplitude for the apologizing opponent (*M* = 3.07 μV, SD = 3.35) was larger than for the non-apologizing opponent (*M* = 1.87 μV, SD = 2.23), *F*(1,22) = 4.27, *p* = 0.051, consistent with our second hypothesis; whereas for high punishment, there was no difference in the amplitude for the two opponents (Figures 5A,C), *F*(1,22) = 0.58, *p* = 0.45. Second, there was a significant interaction between gender and opponent, *F*(1,21) = 14.98, *p* = 0.001 (Figure 5B). Pair-wise comparisons showed that the LPP amplitude for the apologizing opponent (*M* = 3.77 μV, SD = 2.91) was significantly larger than for the non-apologizing opponent (*M* = 2.27 μV, SD = 2.81) among female participants, *F*(1,21) = 13.9, *p* = 0.001, whereas for male participants the amplitude did not significantly differ between the apologizing opponent (*M* = 0.9 μV, SD = 2.75) and the non-apologizing opponent (*M* = 1.65 μV, SD = 2.21), *F*(1,21) = 3.18, *p* = 0.089. Additionally, LPP amplitude for the apologizing opponent was significantly larger among female participants (*M* = 3.77 μV, SD = 2.91) than male participants (*M* = 0.9 μV, SD = 2.75), *F*(1,21) = 7.7, *p* = 0.011, whereas female and male participants’ amplitudes did not significantly differ for the non-apologizing opponent, *F*(1,21) = 0.36, *p* = 0.55.

We tested the correlation between the apology effect on behavior (the difference between the proportion of high punishment for non-apologizing and apologizing opponent) and the difference between the magnitude of LPP when choosing high intensity punishment for the apologizing opponent and the non-apologizing opponent. The correlation was not significant, *r* = 0.041, *p* = 0.85, consistent with the finding in Experiment 1.

***Outcome phase***

*FRN*. FRN (Figure 6A) is more pronounced for negative feedback associated with an unfavorable outcome, such as incorrect response or monetary loss (Gehring and Willoughby, 2002). Therefore, if apology influences FRN responses, we would predict a stronger negativity for loss trials against the non-apologizing opponent than the apologizing one. The three-way ANOVA of gender by opponent by outcome valence revealed that the main effect of opponent was not significant, *F*(1,21) = 0.367, *p* = 0.55. However, the main effect of outcome valence was significant, *F*(1,21) = 22.91, *p* < 0.001, with the mean amplitude for the “loss” trials (*M* = 4.23 μV, SD = 3.35) less positive than for the “win” trials (*M* = 6.24 μV, SD = 3.82). The interaction between gender and outcome valence was significant, *F*(1,20) = 5.65, *p* = 0.028. Females had a larger amplitude for winning trials (*M* = 7.31 μV, SD = 3.9) than for losing trials (*M* = 4.29 μV, SD = 3.65), *F*(1,20) = 31.37, *p* < 0.001, whereas the difference between winning (*M* = 5.16 μV, SD = 1.2) and losing trials (*M* = 4.15 μV, SD = 1.1) did not reach significance for males, *F*(1,20) = 2.45, *p* = 0.133.

*P300*. P300 (Figure 6B) has been shown to be sensitive to valence of rewards (Hajcak et al., 2005). Therefore, we expected that the amplitude would be larger in win trials where the non-apologizing opponent would be punished. The main effect of outcome was significant, *F*(1,20) = 4.53, *p* = 0.046. The mean amplitude for “win” trials (*M* = 12.95 μV, SD = 6.05) was significantly larger than that of “loss” trials (*M* = 11.97 μV, SD = 7.02). The main effect of opponent was not significant, *F*(1,20) = 0.01, *p* = 0.94. The main effect of gender was not significant either, *F*(1,20) = 3.84, *p* = 0.064, nor was the interaction between apology and gender, *F*(1,20) = 2.216, *p* = 0.15.

### Discussion

The behavioral results of Experiment 2 replicated Experiment 1. Both male and female participants selected significantly lower intensity punishments for the apologizing opponent relative to the non-apologizing opponent.

For the decision phase, when participants were presented with the identity of the opponent for whom they would have to select the punishment, ERP showed that the N2 was not altered by apology. However, the amplitude of N2 was altered by punishment intensity. Specifically, its amplitude was larger when participants chose to inflict high punishment to the opponents than when they chose low punishment. This replicates the results from a previous study using a modified version of the TAP, showing that among the higher trait-aggressive participants, selecting high punishments elicited larger N2 than selecting low punishments (Krämer et al., 2008). Therefore, in line with Krämer et al. (2008), N2 in our experiment might be an indicator of aggressiveness.

As for the LPP amplitude during the decision phase, we found two significant interactions. First, choosing low intensity punishment for the apologizing opponent elicited larger LPP than choosing low punishment for the non-apologizing opponent; but no difference was found between the two types of opponents when high intensity punishments were chosen. Second, we found that gender moderated the LPP amplitude between the apologizing and the non-apologizing opponent. Namely, the apologizing opponent elicited a significantly larger LPP among female than male participants, whereas there was no difference between male and female LPP amplitude for the non-apologizing opponent. Third, we found no significant correlation between LPP responses and behavioral punishment. We defer our discussion of these results to the General Discussion.

During the outcome phase, when the result of the reaction time competition was displayed on the screen, FRN and P300 components were only sensitive to outcome valence (Wu et al., 2012) but were not affected by apology or the participant’s punishment choice. Given that no firm conclusion can be drawn from the null effects, these findings will not be discussed further.

## General Discussion

The present study investigated how apology facilitates forgiveness in an interpersonal transgression context. We used an interactive paradigm in which the participant could actively punish two opponents after being passively punished by them. Before he/she had the opportunity to retaliate, the participant received a message from each of the opponents—one apologized for his/her previous behavior and the other one not. Therefore we were able to observe not only the behavioral changes (i.e., the proportion of high punishments selected during retaliation) but also the changes at the cognitive (implicit attitude) and affective/motivational level (ERP) elicited by apology. We discuss the significance of our findings at each of the three levels of analysis and offer a coherent interpretation of such findings, which may help broaden our understandings of the mechanisms of apology and forgiveness.

### Apology Changes Female Victims’ Implicit Attitude Toward the Offender

In Experiment 1, an IAT administrated after receiving the apology and the non-apology messages revealed that the female participants had a more positive implicit attitude toward the apologizing opponent than to the non-apologizing one, although such an effect was not observed for the male participants (Figure 2). The pattern of error rates was consistent with the pattern of the reaction times: for the female participants, responses in the congruent block were both faster and no less accurate than in the incongruent block; for the male participants, responses in the congruent block were both less accurate and no faster than in the incongruent block, indicating that the females had a stronger association between positive concepts and the apologizing opponent.

In accordance with previous studies using only explicit measure of attitude and reactive aggressive behavior, our IAT results confirmed, although only in female, the role of apology in improving victim’s impression of their offender (Ohbuchi et al., 1989; Tabak et al., 2012). Tabak et al. (2012) investigated how apology and conciliatory gestures influence forgiveness. They found that the victims’ perception of their transgressors’ agreeableness mediated the effects of apology and compensation on forgiveness. Importantly, in our paradigm, the participants believed that none of the opponents were aware of the fact that the roles in the game would be switched for the second phase; therefore the apology could not be taken as a strategic move to avoid revenge from the participants. Instead, after being harmed, the expression of remorse and repentance positively changed female participants’ perception of the opponent, as the apologizing opponent might have appeared to be a more trustful and considerate person, relative to the non-apologizing opponent.

Nevertheless, the fact that only female, but not male, participants showed a change of implicit attitude after receiving apology seems to be a challenge to our hypothesis. One possibility could be that in the current experimental setting, the manipulation of apology was not sincere and formal enough: the apologizing opponent did not show up and say sorry directly to the participant. According to Lazare (2004), insincere apology may convey to the victim the transgressor’s indifference to the victim’s loss and suffering, and may amplify the victim’s resentment and aggression toward the transgressor. But the extent to which one finds an apology sincere varies across individuals. It has been demonstrated that compared to men, women judge more often that an apology was deserved (Schumann and Ross, 2010). And thus it is conceivable that the majority of the female participants accepted the apology as sincere, while most of the male participants did not. Another possibility is that the female participants in the current study were more affectively offended by their opponents (cf. Schumann and Ross, 2010), and this might leave more room in women than men for apology to take effects. In other words, women do not only have lower threshold for offense but also have lower threshold for changing their attitude by others’ affective expressions (e.g., apology); men might demand more concrete “actions” rather than just apologizing “words” before they change their implicit attitude toward the offender.

### Neural Substrates of the Effect of Apology on Reactive Aggression

Our electrophysiological results further demonstrate the psychological changes elicited by apology in the victim of interpersonal transgression. Akin to the findings concerning the implicit attitude, the effect of apology on brain responses to the offender was moderated by the gender of the participants: female participants showed higher LPP magnitude, during the decision phase, toward the apologizing opponent than the non-apologizing one, whereas there was no difference in LPP magnitude between the two opponents in male participants. A widely accepted account of the psychological significance of LPP posits that this component reflects the affective and motivational salience of the perceived event/object (Cacioppo et al., 1996; Schupp et al., 2000; Liu et al., 2012), i.e., the importance of the event/object to the survival and welfare of the organism. Along this argument, we could interpret our finding concerning the gender difference in LPP as reflecting that female participants perceive the apologizing opponent as more important than the non-apologizing opponent, and the male participants may just care less about the verbal apology than the female participants. Although this interpretation is based on a relatively small sample size (*n* = 11 and *n* = 12 after splitting into groups) and should be regarded as provisional, it is in line with our IAT results: the verbal apology did not effectively change the male participants’ implicit attitude toward the apologizing opponent.

The LPP magnitude also reflected the differential decision-making processes associated with the apology and the non-apologizing opponents. In this respect, we observed a significant interaction in the magnitude of LPP between apology and the participants’ subsequent punishment choice (Figure 5): in the trials in which the participants chose low punishment, LPP was larger for the apologizing than the non-apologizing opponent. As we pointed out earlier, the LPP reflects the affective and motivational salience of an event/object; the LPP amplitude can be modified by emotion regulation strategies such as reappraisal (Hajcak and Nieuwenhuis, 2006). Thus we suggest that the larger LPP elicited for low punishments to the apologizing opponent (relative to the non-apologizing) is likely to reflect motivational and arousal relevance induced by apology. Forgiveness is often defined as a prosocial motivational change toward the harm-doer (McCullough et al., 2000). The presentation of the apologizing opponent’s portrait at the decision phase might have activated a relatively positive representation encoded in participants’ memory (indicated by larger LPP) and in turn motivated a prosocial response instead of revenge. Consequently, although their mind is set to retaliate after a transgression, viewing the apologizing opponent portrait may have triggered the willingness to forgive and choose lower intensity punishments. The presentation of the portrait of the non-apologizing opponent comparatively did not arouse motivation to reduce punishment intensity in this same context.

Finally, forgiveness requires overcoming the negative feelings prompted by the transgression from an offender (Lazare, 2004). This implies that a dynamic emotion regulation process may underlie apology-induced forgiveness: the victim’s initial response is to retaliate the offender, only at some point of time such initially vengeful motivation is down-regulated by the previously encountered apology. Accordingly, our findings reveal that a relatively early component, N2 (200–280 ms; Figure 4), was not affected by apology during the decision phase. This is consistent with another study using a similar TAP revealed that N2 during the decision phase was related to provocation and indicated aggressiveness (Krämer et al., 2008). Thus, it is possible that apology-induced forgiveness influences later stage processing but not early provocation-related effects. These results lend support to the philosophical notion that reactive aggression, which is a natural tendency, is of greater automaticity and that forgiveness, which is an acquired virtue, is more related to intentionality and continence (Aristotle, 2014).

### Apology Reduces Reactive Aggression Toward the Offender

The behavioral data in both Experiments 1 and 2 revealed that apology reduced the victims’ reactive aggressive behavior, as reflected by the lower punishments chosen for the apologizing opponent than for the non-apologizing one. These findings are consistent with previous studies (Ohbuchi et al., 1989; Strang et al., 2014), and confirm in the laboratory setting the role of transgressor’s apology as a generally effective way to reduce interpersonal revenge and aggression (Lazare, 2004).

However, our experiment distinguishes itself from past research in two main aspects. First, in our experiment, what the participants decided to forgive was an intentional transgressor who had deliberately inflicted harm to them earlier but expressed remorse and apology later on. This feature makes the process of forgiveness in our study closer to the concept of forgiveness in its strictest philosophical sense (cf. Enright and Coyle, 1998). In this regard, our study could make a novel contribution to our understanding of forgiveness beyond the past few previous studies where the object of forgiveness is either unintentional (Young and Saxe, 2009; Yu et al., 2015) or ambiguous offense (Strang et al., 2014). Forgiving an unintentional offense has consistently been associated with the theory-of-mind brain structure (e.g., the temporoparietal junction, TPJ) partly because in that situation counterfactual processing of intention is crucial for forgiveness. In contrast, in our paradigm, apology-based forgiveness relies less on counterfactual processing and more on overcoming anger and adjusting the reactive attitude toward the offender. Second, our major measurements of the impact of apology (i.e., vengeful behavior, ERP, and implicit attitude) did not involve explicit, forced-choice question such as “Do you forgive this opponent?” (e.g., Strang et al., 2014). Instead, we indirectly measured forgiveness by analyzing the proportion of high punishments issued by the participants. This design allowed us to get hold of the psychological processes and neural reactions associated with reception of apology that are closer to real-life situations (Pfeiffer et al., 2013).

### How to Reconcile Our Implicit/Neural Findings With the External Behavior?

It is still an open question as to how the implicit processes (such as those measured by the current IAT and ERP) are related to the explicit behavior. In fact, the exterior behavior, *prima facie*, did not exhibit gender difference: both female and male participants punished the apologizing opponent less than the non-apologizing opponent. For females, this behavioral pattern is consistent with the improvement of implicit attitude (from IAT) and the stronger affective reaction (revealed in ERP) toward the apologizing opponent. The results for male participants, however, did not reveal such a consistent pattern: although they reduced their punishment toward the apologizing opponent, their implicit attitude did not seem to change right after receiving an apology; the latter null effect was also observed on LPP for the apologizing and non-apologizing opponent. Then how could the exterior punishment behavior be reconciled with the implicit measures of attitude and brain activity?

These data from different techniques/modalities might occur at different stages of the psychological processes of forgiveness and probably carry different types of information about such processes. For instance, implicit measures of associations have shown different outcome as compared with explicit measures in past studies and are considered to be more reliable measures of innate, automatic representations and processes (e.g., Phelps et al., 2000). In their seminal work, Phelps et al. (2000) found that racial bias measured by IAT was positively correlated with the strength of amygdala activation to Black-versus-White faces, but not with the direct report of race attitude. This suggests that explicit reports are subjected to controlled inhibition due to external display rules. In a similar vein, in our study male participants behaviorally forgive the apologizing opponent, perhaps due to the demand of social norm or the will for relationship harmony; but they were not actually implicitly/affectively influenced by the apology. Future studies are needed to directly test this hypothesis by, for instance, manipulating the importance/utility of the relationship between the participant and the opponent to the participant (e.g., Nelissen, 2014).

The ERP results seem to support our interpretation. While female participants exhibited larger LPP toward the apologizing opponent, relative to the non-apologizing one, reflecting the salience of apology, male participants did not show such a difference in LPP, indicating that apology did not provoke particular arousal compared to the non-apology. However, similar to female participants, male participants did show a larger LPP when deciding to inflict lower (relative to higher) punishment on the apologizing opponent, while this was not the case for the non-apologizing one. This suggests that although male participants did not care about the informal, verbal apology so much as to allocate more attention to the apologizing than the non-apologizing opponent, they were still pushed in some way to behave more prosocially with the opponent who apologized. In fact, as reported by Bennet and Dewberry (1994), there exist a pronounced pressure to accept apologies even when they are experienced as unsatisfactory (Bennet and Dewberry, 1994).

It is worth noting that the subjective ratings did not show any significant change by apology either in female or male participants (except for the willingness to punish in Experiment 2), in contrast to our behavioral measures (IAT and punishment) and ERP data. This is in line with our argument that forcing participants to express their attitude does not always fit with their actual, implicit attitude or behavior. Thus, our data constitute additional evidence that implicit measures are able to capture psychological reactions that are less/not influenced by social norms, social desirability, or reputation, providing information that are not visible in explicit measures. Clearly, due to the exploratory character of our study, this interpretation stands in a speculative framework. Nevertheless, we believe that our findings open new grounds to a more in-depth understanding of the impact of receiving an apology and forgiveness.

## Conclusion

Taken together, these results provide a novel insight into the psychological processes underlying forgiveness and reception of apology that are not evident in the explicit measures from past studies. Our findings support the notion that expression of remorse from an offender leads the victim to reduce vengeful behavior, either by changing the victim’s implicit attitude toward the offender (particularly for female victims) or by possibly forcing the victim to abide by social norms. We demonstrated that following interpersonal harm, a simple apologizing note from the harm-doer is powerful enough to elicit cognitive, affective, and behavioral changes that underlie the motivation to forgive. Thus, by giving up aggressive and hostile attitude toward a repentant offender, human nature might call for a more harmonious approach of social conflict resolution and, contrary to retaliation mechanisms, apology and forgiveness allow for restoration and maintenance of the relationship.

### Conflict of Interest Statement

The authors declare that the research was conducted in the absence of any commercial or financial relationships that could be construed as a potential conflict of interest.
